# Pathophysiologic Mechanisms of Obesity and Related Metabolic Disorders: An Epidemiologic Study using Questionnaire and Serologic Biomarkers

**DOI:** 10.2188/jea.17.141

**Published:** 2007-09-01

**Authors:** Hiroshi Yatsuya

**Affiliations:** 1 Department of Public Health/Health Information Dynamics, Field of Social Life Science, Program in Health and Community Medicine, Nagoya University Graduate School of Medicine.

**Keywords:** Obesity, Weight Gain, Weight Loss, Insulin, Metabolic Syndrome X, Japan

## Abstract

**BACKGROUND:**

It is still unclear whether individuals with the same degree of obesity but different weight histories since young adulthood have different insulin concentration, prevalence of metabolic syndrome components and their clustering.

**METHODS:**

A cross-sectional study was conducted on 3,399 (for weight difference analysis) and 1,879 (for weight fluctuation analysis) Japanese men aged 40-59 years. Weight difference was calculated by subtracting the recalled weight at about 25 years old from the current weight. The root mean square error around the slope of weight on age (weight - RMSE) was calculated by a simple linear regression model, in which the subject's actual weights at ages 20, 25, 30, 40 years and 5 years prior to the study, as well as current weight, were dependent variables against the subject's age as the independent variable. Each metabolic syndrome component was defined as follows: serum triglycerides ≥150 mg/dL; HDL-cholesterol <40 mg/dL; fasting glucose ≥110 mg/dL; and blood pressure ≥140/90 mm Hg.

**RESULTS:**

Those who gained <10%, <20%, or 20% or more in weight had a significantly higher than unity odds ratio of having two or more metabolic syndrome components in relation to those whose weight remained stable: 1.28 (95% confidence interval: 0.95-1.73), 2.49 (1.91-3.24), and 5.30 (3.97-7.07), respectively. Weight-RMSE was significantly and positively associated with fasting insulin concentration independent of current weight, weight-slope or other lifestyle-related factors.

**CONCLUSIONS:**

Metabolic syndrome components would likely tend to cluster more in individuals with large weight gain on a physiologic basis characterized by high fasting insulin concentration. Furthermore, weight fluctuation was suggested to increase the risk of fasting hyperinsulinemia.

Metabolic syndrome has been proven as a major determinant of ischemic cardiovascular diseases.^1^ Obesity is a central feature of the metabolic syndrome.^2^ However, evidence is not readily available whether individuals who have a similar degree of obesity but different weight histories since young adulthood would have a different prevalence of metabolic syndrome components and their clustering. Furthermore, insulin resistance or hyperinsulinemia has been considered to play a key role in the pathogenesis of clustering multiple cardiovascular risk factors.^3^ We, therefore, examined the association of longitudinal weight histories with fasting insulin concentration as well as metabolic syndrome component and their clustering in Japanese men. Because repeated weight loss and regain is a common phenomenon today due to intentional dieting or unintentional weight change caused by psychological stress,^4^^,^^5^ considerable attention has been paid to the possible deteriorating health effect of weight fluctuation.

## METHODS

### Subjects

To evaluate association between weight differences since about 25 years old and components of metabolic syndrome and their clustering, complete data from two worksites were used (n=3,548). In 1997, participants responded to a self-reported questionnaire including past weight at about 25 years old, medical history and lifestyle characteristics, and participated in an annual health check-up, in which they underwent a physical examination including height and weight measurement, and provided a collection of fasting blood samples.^6^ Those with a medical history of cancer (n=42) and diabetes mellitus (n=107) were excluded. Informed consent was obtained at both worksites. In another analysis to evaluate the association of long-term weight change slope and weight fluctuation with fasting insulin concentration, the weight history data were used from only one worksite on the actual weights at ages 20, 25, 30, 40 years old, and that of 5 years before (in 1992) from the respective health checkup records (n=2,020). Subjects with a medical history of cancer and diabetes mellitus were excluded from the present study (n=88). Finally, the current analysis was restricted to 1,879 men with complete available data on all of the actual weights at ages 20, 25, 30, and 5 years before, together with the serum insulin concentration of the provided blood sample, current weight, and other covariates. The study protocol was approved by the Ethics Review Committee of Nagoya University School of Medicine, Nagoya, Japan.

### Definition of Weight Difference

Weight difference since about 25 years old was calculated for each participant by subtracting the self-reported weight at about 25 years old from the current weight obtained at the 1997 annual health check-up. Current weight and height in 1997 were measured in the morning fasting state to the nearest 0.1 kg and 0.1 cm, respectively, with subjects wearing light clothing and no shoe. Weight difference proportion was calculated by dividing the difference by the weight at about 25 years old. The difference proportion in percentage was categorized into five groups: 20% or more gain, 10-20% gain, 4-10% gain, within 4% (stable), and loss greater than 4%.

### Body Mass Index and Weight Variability Indices

Body mass index (BMI) was calculated as weight in kilograms divided by the square of height in meters and used as the relative weight. Weight variability was divided into two distinct components, trend over time and fluctuation over time, using a simple linear regression model in which each of the subject's five or six actual weights (aged 20, 25, 30 , 40 years old, five years before, and current) was a dependent variable and the subject's ages at examination independent ones. The slope coefficient of this model was used to represent an individual's weight trend of direction and magnitude (weight-slope), while the root mean square error (RMSE) of this model, a standard deviation around this slope, was used to represent the weight fluctuation magnitude (weight-RMSE). The coefficient of variation (CV) of weight is commonly used to describe weight fluctuation, but a person with a slight and steady weight gain over a long period of time can have the same CV as a person with large weight gains and losses with no overall weight gain. As CV and slope are correlated, one cannot discriminate between a nonlinear slope effect and the instability of changes by CV itself. In contrast, RMSE can be a more sensitive measure of instability regardless of the overall trend.

### Definition of Metabolic Syndrome Components and Their Clustering and Measurement of Insulin Concentration

Hypertension was defined as the state with either systolic blood pressure of 140 mmHg or higher or diastolic blood pressure of 90 mmHg or higher. Impaired fasting glucose (IFG) was defined as the state with the serum level of fasting blood glucose of 110 mg/dl or greater. High triglyceride (TG) level was defined as 150 mg/dL or greater. Low high-density lipoprotein cholesterol (HDLC) level was defined as less than 40 mg/dL. In this analysis, clustering of these components was defined as the state in which participants had two or more factors. Serum insulin concentration was measured by solid phase radio-immunoassay (RIABEAD II; Dainabot Co., Ltd., Chiba, Japan).

### Definition of Lifestyle Variables

Smoking status was classified into four levels (never, past, current smoker of 1-24 cigarettes per day, and 25+). Drinking habit was first assessed by the number of drinking days per week (none, 1-3, 4-6, and daily). If present (not none), it was further categorized into three levels by weekly consumption (light, moderate, heavy), that is, daily alcohol consumption times days of drinking per week.^7^ A leisure-time physical activity was assessed by two questions about frequency (seldom, 1-3 times per month, 1-2 times per week, 3 times or more per week) and intensity (vigorous, moderate, light) of the physical activity in their free time. Those who engaged in vigorous activity, 1-2 or more times per week, or moderate activity, more than 3 times per week, were classified as 'regularly active.' Those who engaged in vigorous activity, 1-3 times per month, moderate activity, 1-2 times per week, or light activity, more than 3 times per week, were classified as 'somewhat active.' All others with nonmissing data on these questions were classified as 'not very active.' Vigorous activity was defined in the questionnaire as the level that leaves participants out of breath. Similarly, moderate activity was defined as the level that leaves participants breathing rather hard. Awareness of stress was assessed by the question: 'Do you have much stress in your life?' Participants were asked to select from among four responses: 'very much, ' 'much, ' 'ordinary, ' and 'little.'^8^

### Statistical Analysis

Association between weight difference and component of metabolic syndrome together with their clustering was evaluated by multivariate logistic regression analyses taking stable group as a reference category and adjusting for the age, height, weight at 20 years old, smoking, drinking and physical activity. The associations were presented as the odds ratios (ORs) and the 95% confidence intervals (CIs). Mean fasting insulin levels were calculated for the quartiles of weight-slope and weight-RMSE, respectively. Study-specific quartile cut-points of weight-RMSE were 0-0.52, 0.53-0.97, 0.98-1.59, and 1.60-6.53 kg, and those of weight-slope were −0.54-0.12, 0.13-0.26 0.27-0.40, and 0.41-1.76 kg/year. We statistically adjusted the mean with age at baseline, BMI at age 20 and at baseline, and either weight-slope or weight-RMSE as covariates using a general linear model (Model 2). Further adjustment was also performed with the awareness of stress, smoking, drinking and physical activity status assessed by a baseline questionnaire (Model 3). These variables were entered into the model as covariates as equally spaced ordinal variable (awareness of stress) or dummy variables (others). Insulin concentration is presented as the geometric mean with 95% CI because of the skewness of the data. Differences among means were tested by analysis of covariance (ANCOVA) and post hoc multiple comparisons were performed by the Sidak method. Proportions were tested by chi square test. All reported p values were two-sided, and a p value less than 0.05 was considered statistically significant. Test of linear trend was performed considering quartile categories as equally spaced ordinal ones. All statistical analyses were performed with SPSS^®^ for Windows.

## RESULTS

Prevalence of hypertension, IFG, low HDLC, and high TG in individuals who gained 20% or more weight since the age of 25 were 42.9%, 18.5%, 17.2%, and 46.2%, in contrast to 21.5%, 10.6%, 7.8%, and 18.0% in those whose weight remained stable, respectively (Table 1). The differences in the proportions were significant in all the components (p<0.001, chi square test). ORs of having two or more metabolic syndrome components in those who gained <10%, <20%, or 20% or more in reference to those whose weight remained stable were 1.41 (95% CI: 1.08-1.85), 2.62 (2.06-3.33), and 5.81 (4.46-7.57), respectively (Table 2). Those who lost weight had a significant inverse association with clustering risk factors with OR 0.61 (0.41-0.90).

**Table 1.** Prevalence of metabolic syndrome components according to weight difference categories since about 25 years old.

Weight difference categeries	n	Hypertension*	IFG*	Low HDLC*	High TG*
			
		n (%)	n (%)	n (%)	n (%)
Loss (to −4.1%)	278	60 (21.6)	33 (11.9)	19 (6.8)	42 (15.1)
Stable (−4.0 to 3.9%)	711	163 (22.9)	75 (10.5)	53 (7.5)	119 (16.7)
Gain (4.0 to 9.9%)	701	183 (26.1)	59 (8.4)	67 (9.6)	167 (23.8)
(10.0 to 19.9%)	1,080	327 (30.3)	136 (12.6)	136 (12.6)	370 (34.3)
(20.0+%)	629	271 (43.1)	122 (19.4)	98 (15.6)	286 (45.5)

IFG: impaired fasting glucose; HDLC: high-density-lipoprotein cholesterol; TG: triglyceride.

* : Differences in the proportions of each component were tested by χ^2^ test (all p<0.001).

**Table 2.** Odds ratios of having two or more metabolic syndrome components according to weight difference categories.

Weight difference categories	n	Odds ratio (95% confidence interval)	

		Crude	Multivariate-adjusted*
Loss (to −4.1%)	278	0.82 (0.54-1.25)	0.61 (0.39-0.94)
Stable (−4.0 to 3.9%)	711	1 (reference)	1 (reference)
Gain (4.0 to 9.9%)	701	1.19 (0.89-1.59)	1.28 (0.95-1.73)
(10.0 to 19.9%)	1,080	2.07 (1.61-2.66)	2.49 (1.91-3.24)
(20.0+%)	629	3.72 (2.85-4.85)	5.30 (3.97-7.07)

* : Adjusted for age, height and weight at age 25, smoking status (never, past, current smoker of 1-24 and 25 or more cigarettes per day), drinking (none, light, moderate, and heavy), and physical activity (not very active, somewhat active, and regularly active).

Multivariate-adjusted fasting insulin concentration differed significantly in the quartiles of weight-RMSE and weight-slope (p=0.002 and 0.033, respectively) (Table 3).^9^ The greater the weight-RMSE or weight-slope was, the higher the insulin concentration became accordingly (p for trend <0.001 and =0.033, respectively).

**Table 3.** Serum insulin concentration according to weight-RMSE and weight-slope

		Quartiles of weight-RMSE/weight-sloope									

		Q1		Q2		Q3		Q4		p*	trend p
			
		n	mean (95%CI)	n	mean (95%CI)	n	mean (95%CI)	n	mean (95%CI)		
Weight-RMSE	Model 1^†^		4.2 (4.0-4.4)		4.3 (4.1-4.5)		4.4 (4.1-4.6)		5.0 (47-5.2)^||^	<0.001	<0.001
	Model 2^‡^	474	4.2 (4.1-44)	479	4.3 (4.1-4.5)	459	4.5 (4.3-4.7)	467	4.8 (4.6-5.0)^¶^	0.002	<0.001
	Model 3^§^		4.2 (4.1-4.4)		4.3 (4.1-4.5)		4.5 (4.3-4.7)		4.8 (4.5-5.0)^¶^	0.002	<0.001

Weight-slope	Model 1*		3.1 (2.9-3.2)		4.0 (3.8-4.2)**		4.8 (4.5-5.0)^¶^		6.6 (6.3-6.9)^||^	<0.001	<0.001
	Model 2^†^	480	4.2 (3.9-4.6)	452	4.3 (4.1-4.5)	466	4.4 (4.2-4.6)	481	4.9 (4.5-5.2)^††^	0.050	0.059
	Model 3^‡^		4.2 (3.9-4.5)		4.3 (4.1-4.5)		4.4 (4.2-4.6)		4.9 (46-5.2)^‡‡^	0.033	0.033

CI: confidence interval; RMSE: root mean square error

* : P values by one-way analysis of variance in Model 1 and one-way analysis of covariance in Model 2 and 3.

† : Crude mean

‡ : Age, body mass index at baseline and at age 20, and weight change slope adjusted mean

§ : Age, body mass index at baseline and at age 20, weight change slope, awareness of stress, smoking status (never, past, current smoker of 1-24 and 25 or more cigarettes per day), drinking (none, light, moderate, and heavy), and physical activity (not very active, somewhat active, and regularly active) adjusted mean

|| : significantly higher than Q1, Q2, and Q3 by Sidak method

¶ : significantly higher than Q1 and Q2 by Sidak method

** : significantly higher than Q1 by Sidak method

†† : significantly higher than Q3 by Sidak method

‡‡ : significantly higher than Q2, and Q3 by Sidak method

A similar result was published in reference No. 9.

## DISCUSSION

In the present analyses using longitudinal weight history data, an independent and dose-dependent association was found between weight difference since young adulthood and each metabolic syndrome component and their clustering. Second, weight gain or loss (weight-slope) and weight fluctuation (weight-RMSE) were both associated with insulin concentration. The amount of weight difference was significantly associated with insulin concentration as well (data not shown). Another study of ours had revealed that weight fluctuation was also associated with metabolic syndrome.^10^ These results indicated that risk factors would cluster on the basis of a physiologic background formed or characterized by adult weight gain and weight fluctuation. We have revealed in the present study that weight fluctuation was associated with high fasting insulin concentration independent of current weight and weight gain or loss. Taken together, fasting hyperinsulinemia was suggested to be a common physiologic background from weight gain or weight fluctuation to metabolic syndrome.

Although this is a cross-sectional study in which determination of the cause-effect relationship requires further investigation, several mechanisms by which weight fluctuation would lead to hyperinsulinemia can be considered. First, increased insulin concentration might reflect a change in adiposity. A significant increase in visceral fat mass as a long-term consequence of weight cycling was reported in an experimental study in rats.^11^ Adipose tissues were also found to be significantly enlarged in weight-cycled rats.^12^ Two studies in humans found a positive association of weight fluctuation with waist-hip ratio,^13^ or truncal adiposity.^14^ Further studies using the degree of total or abdominal adiposity are warranted.

Second, a thrifty phenotype,^15^ postulated as an adaptive metabolic dynamic state to a famine-and-feast lifestyle, and characterized as suppressed thermogensis with concomitant skeletal muscle insulin resistance, may explain the present finding of a high fasting insulin level in weight-fluctuating individuals. Co-existing adipose tissue insulin hyper-responsiveness serves to replenish its fat store rapidly when the food becomes available.^15^ The phenotype can be maladaptive in the modern lifestyle of an industrialized society characterized by low physical activity and readily available energy-dense diets so as to exacerbate suppression of thermogenesis and lead to excess adiposity in regaining weight.^15^

As mentioned earlier, the cross-sectional nature of the present study cannot exclude the possibility that the insulin concentration is the cause rather than the consequence of weight fluctuation. Indeed, there have been studies regarding whether or not baseline insulin levels precede weight change, but with inconsistent findings.^16^^-^^18^ Therefore, although the present study has indicated the association between weight fluctuation and higher insulin concentrations, determination of the cause-effect relationship requires further investigation.

The present study used weight fluctuation over a longer period than any other studies, and the measure was based on previously determined weight, as opposed to recalled weight. Associations of disease with weight using self-reported measures are known to result in underestimation because overweight individuals tend to underreport while the underweight tend to overreport.^19^ In addition, the large sample size provided the strong statistical power to detect intercategorical differences.

Weight fluctuation, per se, has been associated with the risk of increased cardiovascular diseases in some prospective studies even after adjustment for the degree of obesity.^20^^,^^21^ These associaions could be explained at least partly by the link between weight fluctuation and high insulin concentration, or with metabolic syndrome.^10^ We have also examined and found a significant and positive association of weight fluctuation with the level of C-reactive protein (CRP), a marker for underlying systemic inflammation,^22^ and speculated that CRP elevation may be another pathway from weight fluctuation to cardiovascular risk. CRP is an acute phase reactant,^23^ a marker for underlying systemic inflammation, and an elevated CRP level has been shown as a strong independent predictor of future cardiovascular diseases .^24^^-^^26^

Altered levels of adipocytokines, such as leptin or adiponectin secreted from excess adipose tissue as a consequence of adult weight gain or weight fluctuation,^27^ may be pathogenically involved in the risk factors' clustering or arteriosclerosis directly. Leptin concentration was found to be linearly associated with white blood cell counts,^28^ a possible marker for future cardiovascular mortality.^29^ Blood pressure level was also related to leptin concentration independent of insulin level,^30^ and decrease in adiponectin level was associated with metabolic syndrome.^31^ Adiponectin concentration was inversely associated with CRP level in extremely healthy individuals, suggesting its anti-inflammatory and anti-arteriosclerotic property.^32^ It might be causally associated with the development of left ventricular hypertrophy.^33^

One of the limitations of the present study may be related to the fact that participants were a series of employees who had been working in a company from around the age of 20, and had been able to keep working until the year 1997. There may have been a group of individuals with large weight fluctuation that had dropped out before the baseline. Thus, the present result may apply only to a relatively healthier population.

In conclusion, from these cross-sectional analyses of middle-aged Japanese men, we found a positive association between the fasting serum insulin concentration and weight fluctuation that occurred from young adulthood even after adjustment for the degree of obesity. The mechanisms for this remain to be solved, and investigations must be undertaken on possible alteration of insulin metabolisms in human by the level of weight fluctuation, and measurement of changes in adiposity are also needed.
